# *Symbiodinium* Transcriptomes: Genome Insights into the Dinoflagellate Symbionts of Reef-Building Corals

**DOI:** 10.1371/journal.pone.0035269

**Published:** 2012-04-18

**Authors:** Till Bayer, Manuel Aranda, Shinichi Sunagawa, Lauren K. Yum, Michael K. DeSalvo, Erika Lindquist, Mary Alice Coffroth, Christian R. Voolstra, Mónica Medina

**Affiliations:** 1 Red Sea Research Center, King Abdullah University of Science and Technology (KAUST), Thuwal, Saudi Arabia; 2 European Molecular Biology Laboratory, Heidelberg, Germany; 3 Department of Anesthesia, UCSF School of Medicine, University of California San Francisco, San Francisco, California, United States of America; 4 Department of Energy Joint Genome Institute, Walnut Creek, California, United States of America; 5 Graduate Program in Evolution, Ecology and Behavior, Department of Geology, State University of New York at Buffalo, Buffalo, New York, United States of America; 6 School of Natural Sciences, University of California Merced, Merced, California, United States of America; American University in Cairo, Egypt

## Abstract

Dinoflagellates are unicellular algae that are ubiquitously abundant in aquatic environments. Species of the genus *Symbiodinium* form symbiotic relationships with reef-building corals and other marine invertebrates. Despite their ecologic importance, little is known about the genetics of dinoflagellates in general and *Symbiodinium* in particular. Here, we used 454 sequencing to generate transcriptome data from two *Symbiodinium* species from different clades (clade A and clade B). With more than 56,000 assembled sequences per species, these data represent the largest transcriptomic resource for dinoflagellates to date. Our results corroborate previous observations that dinoflagellates possess the complete nucleosome machinery. We found a complete set of core histones as well as several H3 variants and H2A.Z in one species. Furthermore, transcriptome analysis points toward a low number of transcription factors in *Symbiodinium* spp. that also differ in the distribution of DNA-binding domains relative to other eukaryotes. In particular the cold shock domain was predominant among transcription factors. Additionally, we found a high number of antioxidative genes in comparison to non-symbiotic but evolutionary related organisms. These findings might be of relevance in the context of the role that *Symbiodinium* spp. play as coral symbionts.

Our data represent the most comprehensive dinoflagellate EST data set to date. This study provides a comprehensive resource to further analyze the genetic makeup, metabolic capacities, and gene repertoire of *Symbiodinium* and dinoflagellates. Overall, our findings indicate that *Symbiodinium* possesses some unique characteristics, in particular the transcriptional regulation in *Symbiodinium* may differ from the currently known mechanisms of eukaryotic gene regulation.

## Introduction

Dinoflagellates are ubiquitous marine and freshwater unicellular eukaryotes. As photosynthetic plankton, they are responsible for much of the primary production of oceans, rivers, and lakes. As photosynthetic marine symbionts, they form mutualistic relationships with reef-building corals and other invertebrates [1]. Approximately half of the 4,000 known dinoflagellate species contain no plastids, and many species are mixotrophic [2]. Dinoflagellates belong to the Alveolata, a large eukaryotic clade that also comprises the ciliates, which are free-living, as well as the Apicomplexans, which all have parasitic lifestyles.

In addition to their ecological diversification, dinoflagellates show some genetic traits that make them distinct from other eukaryotic lineages. In particular, dinoflagellates have extensively methylated nuclear DNA. About 12–70% of thymine bases are replaced by 5-hydroxymethyluracil, and varying levels of cytosine methylation have been observed [3], [4]. Genome sizes are very large and remarkably variable within the group, with estimates ranging from 3–215 gigabases (Gb) in size [5], [6]. The genomic DNA is present in up to several hundred chromosomes per species [7]. Dinoflagellate genomic DNA has been shown to occur in a crystal-like state [8], with chromosomes condensed throughout the cell cycle [9]. Some of these observations initially led authors to conclude that dinoflagellates lacked histones [9]. However, recent genome-enabled studies have confirmed the presence of histones H3 [10], H2A.X [11], and H4 [12] in members of this lineage. Dinoflagellate genomes may host some 40,000–90,000 genes, which might be partly due to high gene copy numbers [13]. Despite the high gene number, dinoflagellate genomes are assumed to consist mostly of non-coding DNA (98–99.9%) [13]. Another unique feature characteristic of the dinoflagellate molecular machinery is the *trans*-splicing of spliced leader sequences [12], [14]. In this process, a highly conserved spliced leader (SL) is transplanted to the 5′ end of mRNAs. SL trans-splicing acts to convert polycistronic mRNAs to monocistronic mRNAs and has also been suggested to regulate gene expression [15].

*Symbiodinium* spp. (Alveolata: Dinophycea) – often referred to as zooxanthellae – are a specific group of dinoflagellates that are intracellular symbionts of many marine invertebrates including scleractinian corals. Although initially considered a single symbiotic species, molecular phylogenetics has uncovered major *Symbiodinium* clades [16] that are separated from each other by tens of millions of years [17]–. Through photosynthesis, *Symbiodinium* algae supply much of their hosts' dietary needs and in return receive shelter, a light-rich environment, and inorganic nutrients [20]. In most cases this symbiotic relationship is reestablished during each host generation [21]. Recent transcriptome-wide efforts have been mainly devoted towards the understanding of the molecular and cellular processes involved in the onset of symbiosis from the host perspective [22]–[25]. From the symbiont's perspective, a relatively small number of ESTs has been analyzed by Leggat et al. [26] and Voolstra et al. [27]. Voolstra et al. [27] compared orthologous cDNA sequences from cultured and symbiotic species (i.e. *Symbiodinium* CassKB8 and *Symbiodinium* C3, respectively), providing some preliminary insight into the genes that might be involved in *Symbiodinium* symbiosis. In a similar fashion, studies have focused on the biochemical and transcriptomic responses to the breakdown of symbiosis (i.e. coral bleaching) in adult corals [23], [27]–[30]. However, thus far there are no comprehensive *Symbiodinium* genome-enabled investigations that can shed light onto the complement of genes associated with susceptibility to bleaching.

In this study, we sequenced EST libraries from two *Symbiodinium* species that are known to establish stable symbioses with coral hosts (clade A: *Symbiodinium* sp. CassKB8 and clade B: *Symbiodinium* sp. Mf1.05b). These data represent the largest dinoflagellate EST data set available to date with more than 56,000 assembled transcripts per species. Annotation of these transcripts yielded new insights into the complex gene repertoire of dinoflagellates, and the mechanisms of nuclear organization of DNA and transcriptional regulation among others.

## Materials and Methods

### Cultures, RNA isolation and sequencing

Two different species of *Symbiodinium* spp., CassKB8 (clade A) and Mf1.05b (clade B), were exposed to a range of different conditions (heat, cold, light, and dark) for 3–6 days to induce expression of a maximum number of genes. Cultures were grown in f/2 medium [31], for Mf1.05b cultures antibiotics were added to the medium to combat bacterial contamination [32]. ‘Hot’ and ‘cold’ cultures were grown at 30–31°C and 19°C, respectively, all other cultures at 27°C. All treatments were subject to a diurnal light cycle (14:10 hrs) of approximately 50 µmol photons/m^2^/s, except the ‘dark’ treatment, for which cultures were grown in darkness for 6 days. For the ‘light’ treatment the light intensity was increased to approximately 120 µmol photons/m^2^/s. Treated *Symbiodinium* were harvested during the exponential growth phase (approx. 10^6^ cells/mL), pelleted and then snap frozen in liquid nitrogen. *Symbiodinium* sp. CassKB8 was originally isolated from *Cassiopeia* sp. in Kaneohe Bay, Hawaii by Robert Kenzie (personal communication). *Symbiodinium* sp. Mf1.05b was isolated from *Montastraea faveolata*, Florida Keys by M.A. Coffroth. Frozen pellets were ground into a fine powder using a pre-chilled mortar and pestle, and powder was added directly to Qiazol lysis reagent (Qiagen, Hilden, Germany). Total RNAs were precipitated with isopropanol, and RNA pellets were washed with 80% ethanol and redissolved in water. Total RNAs were cleaned with RNeasy Mini columns (Qiagen) and pooled in equal amounts for each species. Library preparation for sequencing was carried out differently for both strains. For CassKB8 the RNA was used to construct cDNA libraries using the cDNA Rapid Library Preparation Method as outlined in the Roche kit (Roche 454 Life Sciences, Branford, USA), followed by normalization using the protocol provided for the Evrogen Normalization kit (Evrogen, Moscow, Russia). The normalized dscDNA was then used to construct 454 libraries using the 454 library construction protocol provided in the 454 FLX Titanium Roche kit (Roche, Branford, USA) and then sequenced using the 454 GS-FLX platform. For Mf1.05b cDNA was generated using an oligo-dT primer followed by template switching (Clontech, Mountain View, USA) and subsequently normalized using the same kit as above and sequenced as detailed for CassKB8.

### Data and Assembly

The reads were assembled using version 3.2.1 of MIRA [33] with settings appropriate for transcriptome assembly (–job = denovo,est,normal,454 COMMON_SETTINGS -GE:not = 8 454_SETTINGS -CL:qc = no:cpat = yes:msvs = yes -AS:mrpc = 1 -OUT:sssip = yes:stsip = yes). Adaptors were searched and marked with SSAHA2 [34], and the locations included in the MIRA input files to enable clipping. As MIRA assembles transcripts (not genes), size sorted contigs and singlets were clustered using the UCLUST algorithm as implemented in USEARCH 4.2.66 [35] in both directions with an identity cutoff of 90% in order to estimate the number of genes (Suppl. Table S1). The cutoff was empirically chosen as a conservative estimate to account for sequencing errors and mRNA editing. In the following, clustered contigs and singlets are referred to as genes. To test the effect of clustering on gene families, all contigs belonging to the actin gene family were determined by searching a full length actin sequence from *Symbiodinium* (accession no. AB231899, [36]) against all CassKB8 contigs, and comparing to the clustering of these contigs (Suppl. Table S2). All raw reads are available in the NCBI Short Read Archive (SRA) under the accession numbers SRX076710, SRX076709, and SRX076696. The assembled and annotated sequences are available for download at http://medinalab.org/zoox. In most of the cases, we were not able to identify a SL sequence in our dataset. However, PCRs with a SL and gene specific primer for three genes (actin, Glyceraldehyde 3-phosphate dehydrogenase and β-tubulin) showed that the SL sequence is present in all three genes in CassKB8 (data not shown). Absence of the SL from the transcriptome sequences may be a library preparation or sequencing artifact.

### Annotation

Assembled transcriptome data were annotated as follows: 1) by BLASTX homology search against protein databases, 2) by mapping to pathways using the KEGG annotation service KAAS [37], and 3) by searching for protein domains with InterProScan [38]. The BLASTX homology search was conducted against the Swissprot, TrEMBL [39] and NCBI nr non-redundant protein databases (all as of May 2011) in that order, and the first hit with an e-value below 10^−5^ was retained for annotation. For KAAS pathway annotation and analysis, we used the single-directional best hit (SBH) method to query the set of organisms representative for ‘genes’ as suggested on the KAAS website, with the default bitscore threshold of 60. Determination of completeness of the transcriptome data was also based on the KEGG annotation and manual analysis of the pathways and complexes identified. Protein domains were annotated using the InterProScan software in version 4.6 with all possible applications and in all reading frames [38]. The ‘sig’ and ‘SignalPHMM’ databases were excluded from the InterProScan results, as they do not represent functional protein domains.

### Codon usage

We searched all contigs and singlets against the NCBI nr database using BLASTX to ensure that only codons in the proper reading frame were used to calculate codon usage statistics. For all calculations we extracted and used only the nucleotide sequences corresponding to the best HSP in hits with an e-value of equal or less than 10^−10^. This procedure yielded a total of 4,224,266 and 2,525,073 codons for CassKB8 and Mf1.05b, respectively. Transcriptome data were analyzed for codon usage and the effective number of codons (Nc) [40] with the programs cusp and chips from the EMBOSS package [41]. The maximum number for Nc is 61, which indicates uniform codon usage whereas lower values signify codon bias. We analyzed Nc in relation to the GC content of the third codon position (GC3) through an Nc plot (i.e. a plot of Nc versus GC3s for all genes) to determine whether codon usage heterogeneity exists among different genes in our transcriptome data. In order to look at major differences between genes in relation to codon usage, we performed Correspondence Analysis – a multivariate statistic that displays the greatest variance in codon usage in a two dimensional plot. Correspondence analysis of codon usage was calculated with the software CodonW [42]. One group of transcripts formed a distinct cloud of points in this analysis. In order to analyze this group in more detail, we chose a visual cutoff to separate the member transcripts. We summarized the putative functions for these transcripts by clustering at 90% similarity (as described earlier) and by subsequently counting genes with the same annotation. To ensure accurate results, we counted only transcripts with more than 100 analyzed codons.

### Histones and Nucleosome-Associated Proteins

Histone and histone-associated genes were identified based on gene annotation. Genes were annotated according to the best annotation hit in the corresponding transcript cluster (Suppl. Table S3). Putative histone transcripts with less than 30 amino acids length were excluded from further analysis. Only full-length amino acid sequences of histones (Suppl. Table S3) were considered for phylogenetic analysis. Histone sequences for different H2A, H3 and H3.3 variants were downloaded from the NCBI databases. We preferentially selected sequences from closer and further related species for which more than one histone variant was present. Sequences were aligned using the MUSCLE [43] implementation in Mega5 v.5.05 with standard settings [44]. Phylogenetic trees were reconstructed using maximum likelihood (ML) and Bayesian analysis. ML analysis was performed using the PhyLM v3.0 software [45] available at the “ATGC South of France bioinformatics platform" (http://www.atgc-montpellier.fr). Analyses were performed using the WAG substitution model (as determined by Mr. Bayes mixed model). Tree improvement was assessed using both, Subtree Pruning and Regrafting topological moves (SPR) and simultaneous Nearest Neighbor Interchanges (NNI) algorithms, branch support was assessed via nonparametric bootstrapping using 1,000 replicates. Bayesian analysis was performed using MrBayes v3.1.2. [46] using the following settings: nchains = 4, one cold and three heated chains, with the exception of codon models were two chains were used; the number of steps = generations was set to 1,000,000 with sampfreq = 100 and burnin = 2,500. Convergence was assessed using Tracer v.1.5 [47] and by examining the PSRF values and standard deviation of split frequencies. The best substitution model was assessed using mixed model as recommended by MrBayes and the WAG model was used for subsequent analysis based on the highest posterior probability.

### Transcription factors

We used the comprehensive set of annotated, sequence-specific DNA/RNA binding domains described in [48] to search for transcription factors in our transcriptome data. We included the AP2 domain, which is common in plants, but has recently also been found in apicomplexans [49]. Our analysis was based on Pfam domains with an e-value cutoff of 10^−6^ as provided by HMMER [50] following the approach of Ryu et al. [51]. All contigs and singlets were translated in all reading frames to obtain all possible peptide sequences using transeq from the EMBOSS package [41]. To estimate transcription factor numbers at the gene level, any domain was counted only once 1) per transcript cluster, and 2) per transcript if the transcript contained multiple domains of the same type. In addition, all dinoflagellate ESTs from the NCBI Genbank dbEST database (as of June 2011) were downloaded and analyzed as described above (total number of sequences: 165,532). Finally, all protein sequences from selected outgroup taxa were included in the analysis: *Plasmodium falciparum* and *P. vivax* from PlasmoDB [52], *Paramecium tetraurelia* from ParameciumDB [53], and *Thalassiosira pseudonana*, *Arabidopsis thaliana*, *Drosophila melanogaster* and human from BioMart [54]. Outgroup protein sequences were analyzed with HMMER as described above.

### Antioxidative response

Putative antioxidant genes were identified in a similar manner as the transcription factors. Briefly, we screened our data set for antioxidant-associated genes using a list of pertinent Pfam domains [55] as compiled by Reitzel et al. [56]. We additionally included Pfam motifs for Peroxiredoxin (PF10417), Glutaredoxin2_C (PF04399), Alkylhydroperoxide reductase (PF00578), and exchanged the listed An_peroxidase (PF03098) for peroxidase (PF00141). For outgroup comparisons, we included all protein sequences from *Arabidopsis thaliana*, *Physcomitrella patens*, *Thalassiosira pseudonana* and *Phaeodactylum tricornutum* available through the BioMart database [54]. To estimate numbers at the gene level, domains were counted as previously described in the transcription factor analysis.

## Results

### Transcriptome Data Set

We obtained approximately one million reads of around 400 nt in length from each of the *Symbiodinium* CassKB8 (clade A) and Mf1.05b (clade B) transcriptomes (Table 1). Assembly of the reads yielded 72,152 and 76,284 contigs and singlets for CassKB8 and Mf1.05b, respectively. We clustered all contigs and singlets at 90% identity in order to estimate the true gene number rather than the number of transcripts. This clustering resulted in 57,676 and 56,198 potential genomically encoded genes. The clustering yielded a conservative gene number estimate, as closely related genes from gene families were clustered in one group. For instance, for the actin gene family cluster, 36 contigs clustered into 14 groups with as many as 7 contigs in one group (Suppl. Table S2).

**Table 1:** Overview of the sequencing data, assembly, clustering, and annotation statistics.

	CassKB8	Mf10.5b
**Raw read data**		
No. of useable reads	1,103,642	940,418
Average read length	401	365
Total no. of bases	443,465,967	343,473,807
**Assembly**		
No. of contigs	53,374	48,942
No. of singlets	18,778	27,342
Total bases	61,920,532	45,335,163
Average contig length	1,029	769
**Clustering (90% identity)**		
Clusters (no. contigs and singlets)	8,483 (22,959)	11,407 (31,493)
Unclustered contigs and singlets	49,193	44,791
Total genes estimate	57,676	56,198
**Annotation (percent genes with hits)**		
BLASTX (swissprot, trembl, nr)	41.38%	31.17%
KEGG/KAAS	15.51%	11.10%
InterProScan	34.18%	25.19%

Using BLAST against three protein databases we could only annotate 41% and 31% of all contigs and singlets for CassKB8 and Mf1.05b, respectively. Using KAAS these values were even lower with 15% and 11%. Protein domains could be identified with InterProScan in 34% and 25% of all contigs and singlets (Table 1).

When examining the distribution of hits to the KEGG database in the highest category of the KEGG Brite hierarchy for pathways [57], both transcriptomes showed a similar distribution of genes among categories (Suppl. Table S4). For instance, the highest number of genes had a function in ‘Metabolism’, followed in second place by the ‘Organismal System’ category, and thirdly by the group of genes that are relevant to human diseases. The distribution of genes among these categories and their subcategories is similar to that seen in *P. falciparum*, *P. tetraurelia* and *A. thaliana* (data not shown).

In order to estimate the completeness of our sequenced transcriptomes, we searched the KEGG annotation for components of essential metabolic pathways and protein complexes (Table 2). In addition, we searched for gene families that exist universally in single copy across the tree of life (Suppl. Table S5, [58]). We found the majority of genes for the pathways and complexes analyzed as well as the majority of single copy genes, although the Mf1.05b transcriptome displayed lower gene numbers for the Pentosephosphate pathway, TCA cycle, and the proteasome and spliceosome complexes (Table 2).

**Table 2:** Annotation of pathways and complexes in the transcriptome data (values are numbers of genes, i.e. contigs and singlets clustered at 90% similarity).

Pathway/complex	Known genes	Identified genes	
		CassKB8	Mf1.05b
Glycolysis	10	10	10
Pentosephosphate pathway	7	7	6
TCA cycle	11	10	9
Calvin Cycle	11	11	11
Proteasome	33	31	25
Spliceosome	72	66	63
Universal single copy genes	40	38*	38*

* COG0096 and COG0552 were not identified.

### Codon Usage

GC content values showed a marked difference between both species. The coding GC content in CassKB8 was about 6% higher than in Mf1.05b (Table 3). In particular, values were much lower than previously reported (∼78%) for the third codon position in the dinoflagellate *Alexandrium tamarense* [11], [59], but closer to those reported for the dinoflagellate *Karenia brevis* (53.5%) [60].

**Table 3:** GC content in predicted coding regions of genes with BLASTX e-values<10^−10^.

	CassKB8	Mf1.05b
coding %GC	56.41	50.57
3rd position %GC	68.90	54.96
Nc*	51.36	55.56
No of codons	4,224,266	2,525,073

* Nc = number of effectively used codons.

**Figure 1:**
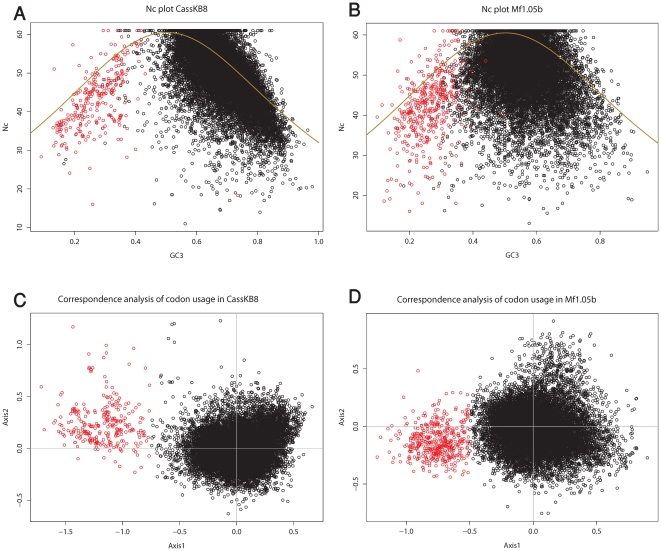
Nc and correspondence analysis of codon usage plots. (A, B) Plots of the effective number of codons (Nc) plotted versus third codon position GC content (GC3) in CassKB8 and Mf1.5b respectively. The red points are the same genes as in C and D, respectively. The yellow line represents the neutral expectation for Nc. (C, D) Correspondence analysis of codon usage. The genes separated from the main cloud are marked red.

The analyzed *Symbiodinium* species show some codon bias with Nc values of 51.36 for CassKB8 and 55.56 for Mf1.05b, respectively. In comparison, codon bias is higher in *A. tamarense* with 43.64 [59]. In the Nc plots (Figure 1 A, B), the absence of codon usage bias as a null hypothesis (Nc_H0_) is displayed as a solid curve [40], and genes which lie below this line have a stronger codon bias than expected based purely on their GC3. In both species most genes have an Nc value lower than Nc_H0_, indicating codon bias and that codon usage is not determined by GC content alone (GC3) (Figure 1 A, B).

The distribution of genes on the two axes on the correspondence plots (Figure 1 C, D) showed one cluster of genes around zero on both axes, and a secondary cluster of genes offset on axis 1. To separate these ‘outlier’ genes, we visually chose a cutoff of < = −0.75 for CassKB8 and < = −0.5 for Mf1.05b, which yielded 270 and 431 genes, respectively. The genes in these separated clusters have much less GC in the third codon base than the majority of genes in both species. Most of these contigs and singlets represented genes encoded by the chloroplast genome, which has been shown to exist in the form of short circular DNA molecules, termed minicircles, in peridinin-containing dinoflagellates such as *Symbiodinium* [61], [62] (Table 4). In addition to chloroplast genes, the list includes cytochrome oxidase subunit 1, a mitochondrial gene, and single copies of a diverse group of other genes that did not seem to be related to each other in function.

**Table 4:** Genes that are outliers in the correspondence analysis of codon usage (red points in Fig. 1).

	Location	CassKB8	Mf1.05b
photosystem II protein D1 (psbA)	C	12	20
photosystem II CP47 protein (psbB)	C	25	17
cytochrome b6 (petB)	C	3	10
ATP synthase subunit alpha (atpA)	C	1	9
photosystem II CP43 protein (psbC)	C	7	7
ATP synthase subunit beta (atpB)	C	5	6
photosystem II protein D2 (psbD)	C	1	5
cytochrome b6/f complex subunit 4 (petD)	C	5	3
cytochrome oxidase subunit I (COX1)	M	3	2
Peptide-N(4)-(N-acetyl-beta-glucosaminyl)asparagine amidase	N	0	1
Histidinol-phosphate aminotransferase	N	0	1
Probable cysteine desulfurase	N	0	1
Ribosomal RNA small subunit methyltransferase B	N	0	1
Type I iodothyronine deiodinase	N	0	1
Ureide permease 1	N	0	1
Ankyrin repeat and SAM domain-containing protein 6	N	1	0
Collagen alpha-1(I) chain	N	1	0
Sensor protein degS	N	1	0

The assumed cellular location is noted as follows: C, chloroplast minicircles; M, mitochrondrium; N, nucleus. All genes were grouped according to their BLASTX annotation and the number of genes for each annotation is shown for both species. Genes with less than 100 analyzed codons were not included.

### Histones and Nucleosome-Associated Proteins

The absence of histones was in the past perceived as one of the peculiarities of dinoflagellate genetics. Recent analyses of diverse dinoflagellate ESTs, however, revealed nucleosome components, including representatives of the four nucleosome core histones [14]. We have found a total of 20 histone-encoding genes, 53 histone-modifying enzymes as well as several nucleosome- and chromatin-remodeling associated genes in both *Symbiodinium* transcriptomes (Table 5). *Symbiodinium* sp. CassKB8 contains several copies of each of the four core histones H2A, H2B, H3, and H4. Histones H2A, H2B, and H3 include members of more than one subfamily, such as orthologs of the minor histone variants H2A.Z as well as putative H3.3 and H3.4 orthologs (Table 5). In Mf1.05b, we found three H2A.Z-like transcripts but no H2A.X ortholog. H3 was represented by two genes similar to the H3.3-like minor histone and a H3-like centromeric protein CSE4. Only one copy of histone H4 and none for H2B were detected in Mf1.05b (Table 5).

**Table 5:** Comparison of histones and nucleosome-associated proteins from this and previous studies (DinoEST).

	CassKB8		Mf1.05b		DinoEST		Study
H2A	3		3		2		
H2A.X		2		0		2	Lin et al 2010 [14]; Sanchez-Puerta 2007 [63]; Hackett 2005 [11]; this study
H2A.Z		1		3		0	this study
H2.B		0		2		Lin 2010 [14]	
H2B.2		1		0		na*	this study
H2B.4		1		0		na*	this study
H3		3		3			Okamoto 2003 [10]; Leggat 2007 [26]; Lin 2010 [14]; this study
H3.3		2		2		na*	this study
H3.4		1		0		na*	this study
H3-like CSE4		0		1		na*	this study
H4	3		1		1		Lin 2010 [14]; this study
Histone acetyltransferases	2		4		0		this study
Histone deacetylation	5		8		2		Lin 2010 [14]; this study
Histone methylation	9		15		1		Lin 2008 [117]; this study
Histone demethylation	5		5		0		this study
Histone associated	3		2		0		this study
Nucleosome assembly	2		3		1		Lin 2010 [14]; this study
Chromatin remodeling	11		9		0		this study

* Subtype not specified.

**Figure 2:**
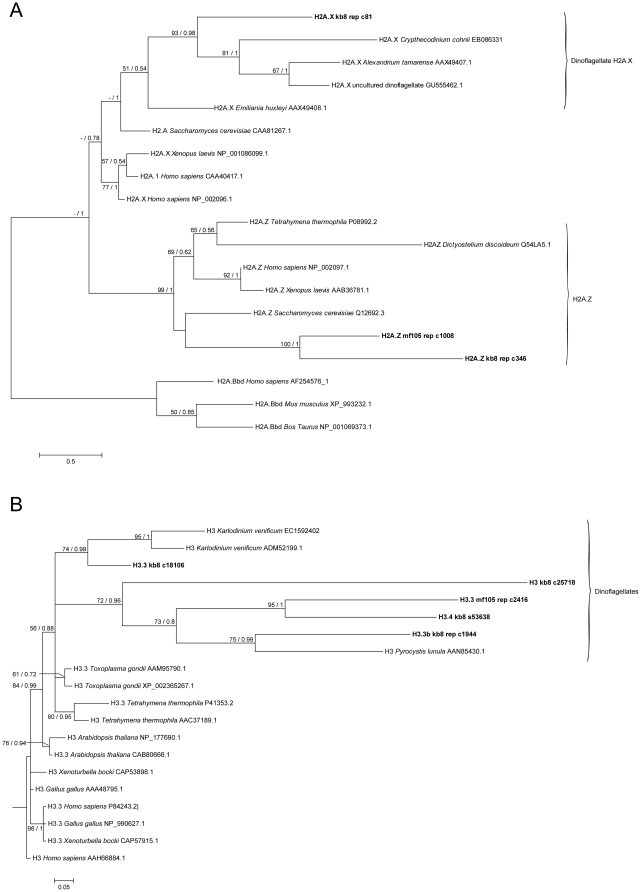
Phylogenetic analysis of histone sequences. H2A- and H3-like sequences from *Symbiodinium* sp. CassKB8, Mf1.05b, and other organisms were used to calculate phylogenetic trees. The trees were inferred using contigs and singlets with full-length amino acid sequences of (A) H2A and (B) H3-like genes using Maximum-Likelihood and Bayesian analysis. Bootstrap values and posterior probabilities are provided as ML/MB for nodes with support above 50% or 0.5. The singlet and contig names are provided for *Symbiodinium* sp. CassKB8 and Mf1.05b sequences (in bold), other taxa are shown as species name followed by GenBank accession number. The H2 tree was rooted for H2A.Bbd sequences whereas the H3 tree was rooted for *Homo sapiens* H3.

Phylogenetic analysis of H2A-like full-length sequences grouped with strong support one of the identified CassKB8 genes with the previously identified dinoflagellate H2A.X sequences from *Alexandrium tamarense* [11] and *Crypthecodinium cohnii* [63] (Figure 2A). The classification of this genes as of dinoflagellate origin was further confirmed by the presence of the H2A.X signature motif ‘SEQY‘ in the full-length sequence encoded in the contig kb8_rep_c81. In general H2A.X sequences did not cluster by variant. This was expected since H2A.X genes are known to have arisen multiple times during evolution of the H2A gene family [64], [65]. In contrast to that, the *Symbiodinium* H2A.Z-like sequences were clearly separated from the H2A.X sequences and formed a group with the H2A.Z sequences from other species, thus reflecting the single evolutionary origin of the H2A.Z protein [64]. The histone H3 family is a diverse histone family [66]. In line with that, we found the highest number of gene copies for H3-like histones. Phylogenetic analysis of the putative *Symbiodinium* H3 genes places them within well-supported dinoflagellate H3 histone clades (Figure 2B) [64]. However, the putative H3 genes identified here cannot be clearly classified into subfamilies based on phylogenetic grouping since the different variants do not resolve into distinctive groups as is the case for H2A.Z (Figure 2B). This is expected as the different H3 variants evolved multiple times independently in different lineages, including plants, animals, ciliates and apicomplexans [64].

Apart from the nucleosome core histones, we identified a variety of histone-modifying proteins including histone acetyltransferases, deacetylases, methylases, and demethylases as well as several nucleosome assembly and histone binding proteins in both species (Table 5). Furthermore, we found the histone-associated chaperone ASF1 in CassKB8 and the Chromatin assembly factor 1 (CAF1) in Mf1.05, which have important roles in chromatin transactions [67], [68]. We found more histone-modifying genes in Mf1.05b than in CassKB8, 32 and 21 genes, respectively. Histone methylases appear to be the most common type of histone-modifying proteins in both species, followed by deacetylases and demethylases (Table 5).

### Transcription factors in *Symbiodinium*

While histones take part in gene regulation at the genome level, the most important proteins that influence transcription of individual genes are transcription factors (TFs). We found a low number of such domains in *Symbiodinium*. In the whole dataset, only 156 and 87 genes contained at least one known protein domain for sequence-specific DNA-binding activity in CassKB8 and Mf1.05b, respectively. These numbers correspond to only 0.27% and 0.15% of all genes (as determined from clusters at the 90% similarity level) (Table 6). A similar result was obtained when the same analysis was conducted on the collection of all dinoflagellate sequences available in the Genbank EST database dbEST, with a percentage of 0.29% of all clustered EST sequences containing at least one transcription factor domain.

**Table 6:** Number of transcription factor domains found in *Symbiodinium* genes (based on 90% similarity clustering of contigs and singlets) and of all dinoflagellate ESTs available in Genbank dbEST.

	CassKB8	Mf1.05b	All dino ESTs from dbEST
No. of genes with transcription factor domain	156	87	272
Total no. of genes with Pfam annotation	18,564	13,495	24,098
% contigs with transcription factor domains of all Pfam annotated	0.84	0.64	1.13
Total no. of clusters	57,676	56,198	92,308
% contigs with transcription factor domains of all clusters/genes	0.27	0.15	0.29

**Figure 3:**
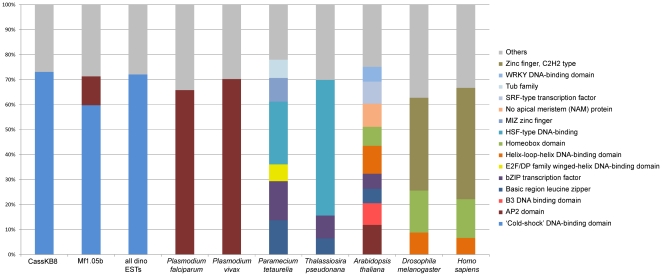
Transcription factor domain composition. The relative fraction of the most abundant transcription factor domains in the *Symbiodinium* transcriptomes, all dinoflagellate ESTs from the NCBI dbEST database, and other eukaryotes. Searches were performed by using HMMER to search domain models for DNA binding domains, with an e-value cutoff of < = 1e−6. Domains which make up less than 5% were grouped in the ‘others’ category.

Not only is the overall number of TF domains low, but the distribution of domains was also different than in other organisms. For instance, Zinc finger C_2_H_2_ domain TFs, which make up the largest fraction of TFs in many eukaryotes such as human and *Drosophila*, were completely absent from the dinoflagellate sequences analyzed here (Suppl. Figure S1). The distribution of the most common TF domains is distinct from the apicomplexans *P. falciparum* and *P. vivax*, the ciliate *P. tetraurelia*, the heterokont diatom *T. pseudonana*, the plant *A. thaliana*, and the insect *D. melanogaster* as well as from human (Figure 3). The most common domain in *Symbiodinium* was the ‘cold shock factor’ DNA-binding domain, making up more than 60% of the transcription factor domains of CassKB8 and Mf1.05b. This domain is a β-barrel domain present in most organisms from all three domains of life. This type of transcription factor also appeared to be among the most common in all dinoflagellates as assessed from dbEST (Figure 3). This domain also occurs in all non-dinoflagellate species studied, though only a few genes contained it (Suppl. Table S6).

### Antioxidative response

Given the importance of Reactive Oxygen Species (ROS) in the bleaching-associated breakdown of the symbiotic relationship between *Symbiodinium* and their coral host, we screened our data for genes associated with the antioxidative response. We used a Pfam protein domain-based approach to assess the antioxidant gene repertoire in both *Symbiodinium* species as well as in four photosynthetic outgroup taxa for which whole genome data were available, namely the land plant *A. thaliana*, the bryophyte *P. patens*, and the diatoms *T. pseudonana* and *P. tricornutum*. We chose plant species for the comparative analysis because land plants are known to possess an efficient antioxidant enzymatic machinery, which allows them to deal with extreme climates and stresses [69], [70]. *T. pseudonana* and *P. tricornutum*, in turn, represented outgroup species more closely related to dinoflagellates that share a similar marine lifestyle with dinoflagellates.

The *Symbiodinium* transcriptomes encoded higher numbers of some proteins involved in the antioxidative response when compared to plants and diatoms, specifically, those containing the Nickel-containing SODs (Sod_Ni), Thioredoxin (Trx), and glutaredoxin 2 (Grx2) domains (Table 7). Interestingly, in contrast to plants, CassKB8 and Mf1.05b possess Sod_Ni, which are common in prokaryotes. Four of the Sod_Ni encoding genes in CassKB8 (kb8_rep_c6308, kb8_rep_c17584, kb8_rep_c1869 and kb8_rep_c6458) and five in Mf1.05b (mf105_rep_c13460, mf105_rep_c40857, mf105_rep_c42288, mf105_rep_c543 and mf105_s69277) were annotated as being Ubiquitin orthologs based on BLASTX. A protein domain analysis confirmed that these genes encoded both a conserved Sod_Ni and an ubiquitin domain. Due to this unexpected result, we searched our sequences against the NCBI nr and dbEST using BLAST to analyze whether this domain composition was restricted to a certain set of species. We found genes encoding both domains in eukaryotic lineages such as the stramenopiles *Phaeodactylum tricornutum* (XP_002183736), *Chaetoceros neogracile* (EL622395) and *Aureococcus anophagefferens* (EGB03009), as well as in different dinoflagellate species including *Karlodinium brevis* (EX871806), *Karlodinium micrum* (EC161447), *Karlodinium veneficum* (GH269044), and *Heterocapsa triquetra* (EU153190) where these genes appear to be common. The only eukaryotic genes outside the chromalveolates displaying this domain signature were found in *Micromonas* sp. (XM_002506486, XM_003063226), and none were found in prokaryotes.

**Table 7:** Comparison of the antioxidant gene repertoire between *Arabidopsis thaliana*, *Phycomitrella patens*, *Symbiodinium* sp. CassKB8, *Symbiodinium* sp. Mf1.05b, *Thalassosira pseudonana*, and *Phaeodactylum tricornutum* based on Pfam domains associated with antioxidant function.

Function	Type	PFAM	*A. thaliana*	*P. patens*	CassKB8	Mf1.05b	*T. pseudonana*	*P. tricornutum*
Sod_Cu	Superoxide dismutase	PF00080.14	4	7	3	0	0	1
Sob_Fe_N	Superoxide dismutase	PF00081.16	5	4	4	2	4	2
Sod_Fe_C	Superoxide dismutase	PF02777.12	5	3	5	6	3	2
Sod_Ni	Superoxide dismutase	PF09055.5	0	0	5	10	0	1
Catalase	Catalase	PF00199.13	3	7	0	0	0	1
Peroxidase	Peroxidase	PF00141.17	82	65	27	24	16	10
GSHPx	Glutathione peroxidase	PF00255.13	9	4	5	1	2	3
Thioredoxin	Thioredoxin	PF00085.14	79	70	106	73	55	41
Glutaredoxin	Glutathione reductase	PF00462.18	52	28	29	17	13	11
Ferritin	Ferritin	PF00210.18	6	4	2	0	0	1
1-cysPrx_C	peroxiredoxin	PF10417.3	3	4	4	2	1	2
Glutaredoxin2_C	Glutaredoxin2_C	PF04399.7	0	0	2	5	1	1
AhpC-TSA	Alkylhydroperoxide reductase	PF00578.15	45	43	28	18	19	22

The thioredoxin (Trx) superfamily comprises different groups of proteins that share a common structural motif. These include thioredoxins (Trx) and protein disulfide isomerases (PDI) as well as glutathione peroxidases (GSHPx) and glutaredoxins, the last two of which are represented separately in this study and are therefore not addressed as Trx here. Comparison of genes encoding putative Trx domains across the six species analyzed here revealed an unexpected high number of genes in both *Symbiodinium* species (Table 7). In CassKB8 we identified a total of 106 genes encoding a Trx domain, which is substantially higher than what is found in the plants *Arabidopsis thaliana* and *Physcomitrella patens* (79 and 70), while 73 putative Trx genes were identified in Mf1.05b. This result is in stark contrast to the comparably low number in the diatoms *Thalassiosira pseudonana* and *Phaeodactylum tricornutum*, where only 55 and 41 Trx domain encoding genes appear to be present in the genomes (Table 7).

## Discussion

### Assembly and completeness

The sequence data reported in this study comprises the largest transcriptome of dinoflagellates to date, and surpasses the available number of dinoflagellate sequences currently in public databases. The number of genes that can be estimated from the data is around 56,000, more than twice the number of genes predicted for the human genome. However, this number is not too far from the 40,000 estimated genes in *Symbiodinium* genomes, based on genome size and its correlation with gene number [13]. One caveat with shotgun sequenced transcriptome data is the possibility of assembling fragmented transcripts. These would artificially increase the gene count, a general problem that is hard to evaluate in a transcriptome where most genes do not have orthologs in fully sequenced genomes. However, our gene number estimate is similar to an estimate based on Illumina sequencing data with much higher coverage (unpublished data), which yields more than 43,000 genes of 500 bp and larger. Furthermore, it is known that dinoflagellates have large gene families with some very closely related members [71], [72]. Such closely related genes may be grouped together in the clustering process performed for this study. In the example tested here, the actin gene family in CassKB8 has 36 contigs and singlets as members, which were grouped into only 14 clusters. Thus, this method makes our gene number estimate more conservative.

As found in other dinoflagellate sequencing data sets, the majority of transcripts do not have similarity to sequences in GenBank or KEGG [11], [14], [27], [60]. This novelty could be expected of an organism which is evolutionary distant from most model organisms. Most of the KEGG-based annotations (10–15% of all genes) fall into the metabolic pathways category. The completeness analysis showed that the majority of the standard “housekeeping" genes in the pathways and complexes are present. The difference between the two species, namely the lower coverage in Mf1.05b, probably arose due to the differences in the sequencing read length, which influences the assembly process and may reflect the different sequencing library generation protocols. In addition, all but two of the genes that belong to universal single-copy gene families were found. Ribosomal protein S8 (COG0096) and signal recognition particle GTPase (COG0552) were not identified in either of the two species. Although this absence is not conclusive without the availability of completely sequenced genomes, it will be interesting to see whether and when a lineage-specific loss of these gene families has occurred.

The genus *Symbiodinium* is comprised of a large number of species encompassed in nine major lineages (clades A–I) [17], [73]. *Symbiodinium* spp. are crucial components of coral reef ecosystems as endosymbionts of corals and other marine invertebrates. However, few analyses exist which identify genes that might play a role in physiological differences, e.g. susceptibility to bleaching of the different symbiont species or clades [26], [27]. Using two *Symbiodinium* species, it becomes possible to conduct an evolutionary screen for candidate adaptive genes involved in symbiosis as has been recently conducted for two coral species – i.e. *Acropora millepora* and *A. palmata* [74], [75]. Identifying adaptively evolving genes – via the ratio of the relative rates of synonymous and nonsynonymous substitutions (*d_N_/d_S_*) of ortholog genes, [76], [77] can be a powerful strategy to narrow gene lists to a few candidates. However, the synonymous nucleotide changes per synonymous site (*d_S_*) we calculated for the two species analyzed here far exceeded 1 (avg *d_S_* 26.3 as estimated by PAML, a value that gets increasingly inaccurate if *d_S_*>1), indicating that multiple substitutions may have occurred at a single site. For this reason, we did not conduct an evolutionary analysis. Nonetheless, having the benefit of two transcriptome data sets that were sequenced at similar depths, we were able to independently confirm all of our findings in both *Symbiodinium* species. In this regard, our estimate of gene numbers, the paucity of transcription factors as estimated from DNA binding domains, the presence of full sets of histones, etc. seem to be general hallmarks of *Symbiodinium* biology rather than clade- or species-specific adaptations. Many of these questions will be answered more definitively in the near future as several whole genome *Symbiodinium* sequencing projects are currently underway.

### Codon usage

We estimated the GC content of the third codon position from a large number of codons in our dataset. GC3 has been found to be a good predictor of the overall genomic GC content [78]. EST data for dinoflagellates in the literature already suggest that GC content in the third codon position is highly variable, from 50% to 77% [11], [59], [60]. It is surprising, however, that we find a large difference of about 14% in GC3 also in two species within the genus *Symbiodinium*. It is usually assumed that differences in GC content between species stem from genome wide mutational bias [79]. If this is true, and if GC richness is homogeneous across the genome, then it stands to reason that there are indeed different mutational mechanisms at work across even closely related dinoflagellate species.

GC content also influences codon usage bias, in addition to a range of other factors such as mutational bias, selective pressures depending on expression strength, abundance of tRNA genes, and environmental factors, and can also be a means of transcriptional control (see [80] for review). Both *Symbiodinium* species have a more relaxed codon bias than *A. tamarense*. Codon usage bias is often coupled with growth rate, as not all tRNAs are present in the cell in equal amounts [81]. Accordingly, the variable codon bias detected between *Symbiodinium* sp. CassKB8 and Mf1.05b may reflect physiological differences (e.g. growth rate, thermal tolerance).

The effective number of used codons is mostly dependent on the overall GC value in the coding sequences. It is shifted towards lower values in Mf1.05b, as this strain has a lower overall GC content. Overall the picture looks similar to the one found previously for *A. tamarense* [59], where the majority of genes are below the Nc expected for neutral evolution of codon usage. This suggests that these genes may underlie selective processes that favor certain codons, as is known from highly expressed genes. The correspondence analysis of codon usage (Figure 1 C, D) shows a group of genes that is separated from the main cloud, almost all of these are encoded in the chloroplast and mitochondrial genomes. The chloroplast genome in dinoflagellates deviates from the organization that is found in most plants and algae; many genes seem to have transferred to the nucleus, and the genes that are still present in the chloroplast are encoded on several small DNA molecules, termed minicircles, that contain only one or a few genes (see Lila Koumandou et al. [82] for review). Codon usage in these genes has been found to be different than in nuclear genes, and some contain unusual start codons [62]. Most of the genes that are known to reside on minicircles are grouped together with those genes whose codon usage deviates from the norm. However, none of the chloroplast genes that have been found to be transferred to the nucleus in dinoflagellates are in that group. This supports the data presented in the literature so far, and makes it unlikely that more genes remain encoded in the chloroplast minicircles than currently known for peridinin-containing dinoflagellates, including the *Symbiodinium* species analyzed here.

### Histones and Nucleosome-Associated Proteins

Until now, no complete set of the nucleosome core histones has been identified in a single dinoflagellate species. Here, we report the complete set of nucleosome core histones as well as the minor histones H2A.Z and several H3 variants in a single dinoflagellate species (*Symbiodinium* sp. CassKB8). Our results indicate that dinoflagellates possess not only a basic nucleosome machinery, but also specialized histones that are known to be involved in transcriptional and epigenetic regulation, e.g. H2A.Z [83]–[85] and H3.3 [64], [86], [87].

In contrast to the histones H2B and H4, the histones H2A and H3 have highly conserved ubiquitously expressed variants with specialized functions. H2A.Z is associated with the promoter region of actively transcribed genes linked to transcriptional competence [83], [84], and is also involved in epigenetic regulation [85]. Studies in *A. thaliana* have shown that DNA methylation and H2A.Z incorporation are mutually exclusive. DNA methylation plays a pivotal role in establishing and maintaining an inactive state of a gene, suggesting that the transcriptional activity promoted by H2A.Z might be conferred through the inhibition of DNA methylation [85]. This is in agreement with studies on *Amphidinium carterae* and *Symbiodinium microadriaticum*, whose genomes appear to be hypermethylated [88]. Furthermore, it has been shown that the methylation status of important photosynthesis genes is correlated with their transcriptional activity [89]. Hence, dinoflagellates might be able to use the nucleosome machinery for transcriptional regulation through the regulation of the methylation status of specific loci. The variation we observed in *Symbiodinium* H3-like proteins is also suggestive of the role of the nucleosome machinery in transcriptional regulation, with possible subfunctionalization of the multiple variants. Finally, we found that *Symbiodinium* species contain various genes for the modification of histones including methylation and acetylation as well as orthologs of the histone-specific chaperones ASF1 and CAF1. ASF1 is involved in the modulation of local chromatin structure during gene-selective silencing in *Drosophila* [67], whereas CAF1 is mainly associated with processes involving DNA, such as DNA replication and DNA repair [68].

Overall, our results provide corroborating evidence for the presence of a functional nucleosome machinery in dinoflagellates. We found specialized histones and histone-associated proteins that are known to be involved in transcriptional and epigenetic regulation. Given that histones appear to be rare in the dinoflagellates nucleus [14], [90], one might speculate that dinoflagellates may employ the nucleosome machinery for transcriptional regulation rather than chromatin packaging. In that case, one would expect that the transcriptional regulation of genes is mainly regulated at the level of accessibility thereby generically switching transcription on or off. Consequently, activated loci would likely display very similar transcription levels. Previous studies have shown that transcriptional levels across many genes indeed appear to be similar [14], [91], [92].

### Transcription factors in *Symbiodinium*

The unusual chromatin structure, low concentration of proteins in the nucleus, and the very large genomes of dinoflagellates raise the question whether gene regulation is realized with the same mechanisms as in other eukaryotes. Transcriptional regulation might play a minor role in dinoflagellates as opposed to other mechanisms of regulation. Here, we analyzed the number and composition of transcription factors in *Symbiodinium* to get a better understanding of gene regulation. We identified only a small number of proteins with sequence specific nucleic acid binding domains (i.e. putative transcription factors). Transcription factors have been shown to scale with genome size [93], and make up 6–9% of all genes of higher eukaryotic transcriptomes (Suppl. Table S7). The percentages found here for dinoflagellates are much lower than those for other protists such as *Plasmodium*, even though *Plasmodium* has a reduced genome due to its parasitic lifestyle.

The assemblage of transcription factors in *Symbiodinium* seems to be completely different from other eukaryotes; common domains such as zinc fingers, helix loop helix, AP2, or homeobox domains are rare or absent. This is also true for the other dinoflagellates represented in the set of ESTs analyzed here, as the set of transcription factor domains and their abundances are quite similar to those found in *Symbiodinium*. This low abundance of transcription factors appears to be a genomic signature of the dinoflagellate clade.

A similar conclusion about an apparent low number of transcription factors in *P. falciparum* and apicomplexans in general [94], [95] was challenged through the discovery of the ApiAP2 family of transcription factors in this group [49]. The 27 members of the ApiAP2 family in *P. falciparum* fill the gap of the apparent lack of transcription factors, and many have now been investigated in detail and shown to be conserved the Apicomplexa [96]. It seems plausible that dinoflagellates may also contain yet undescribed transcription factor families that would represent part of the “missing" transcriptional regulatory machinery. Thus, it is interesting that more than 60% of the putative transcription factors identified carry a ‘cold shock’ domain (CSD). This domain is not very common in other eukaryotes, suggesting a lineage-specific expansion in dinoflagellates. Such lineage-specific expansions of different transcription factor domains have been found in multiple taxa throughout the tree of life [97], [98]. Originally identified as a reaction to cold shock in *E. coli*, proteins with CSD domains have now been associated with a wide range of functions. They can act as transcription factors by binding DNA (i.e. Y-box factors), but many interact with RNA rather than DNA. They are involved in regulation of transcription, splicing, and translation, and influence mRNA stability as RNA chaperones (see [99] for review). This observation fits with the notions that 1) regulation in dinoflagellates may take place after transcription, and 2) that RNA editing is widespread [100]. Thus, proteins with cold shock domains may be responsible for much of the transcriptional regulation in dinoflagellates. Considering that *Symbiodinium* undergoes a dramatic change in its environment and lifestyle upon entering invertebrate hosts, a need for efficient regulation of a large number of genes might be advantageous. However, as all data gathered here are based on *Symbiodinium* grown in cultures, it is possible that more or different types of transcription factors are expressed in the symbiotic state. As *Symbiodinium* genomes are currently being sequenced, this question can be conclusively answered in the near future as the genome sequence becomes available.

### Antioxidative response

The impact of ROS on the symbiosis of *Symbiodinium* and its marine invertebrate hosts is likely to affect mechanisms to cope with photosynthesis-generated ROS in order to prevent the breakdown of the symbiotic relationship. Our analysis shows that *Symbiodinium* also possesses a rich antioxidant gene repertoire, but surprisingly appears to lack or transcribe below detection limit the enzyme catalase (note that catalase activity has been shown by Merle et al. [101]), one of the central enzymes in eukaryotic cellular redox-chemistry. In contrast, catalase was among the most abundant transcripts in *Aiptaisa pallida*, an anemone host for *Symbiodinium* [102]. However, since the transcriptome sequences analyzed here were derived from cultured zooxanthellae it cannot be excluded that the catalase gene is only expressed *in hospite*. One of the main differences between *Symbiodinium* and diatoms or plants is the presence of several prokaryotic Ni-type SODs in both *Symbiodinium* species, which were not present in the plant species and were only represented by a single gene in the diatom *T. pseudonana*. The presence of bacterial proteins is not surprising given that lateral gene transfer between prokaryotes and eukaryotes, especially protists, is common [103], [104]. Furthermore, several genes of bacterial origin have already been identified in *Symbiodinium* [26]. To our surprise, we found that some of the Ni-type SOD genes identified here also encode an additional ubiquitin domain. The ubiquitin domain is a 76 amino acid domain found in eukaryotes, whereas the SOD_Ni domain is supposedly of prokaryotic origin [105], suggesting that these transcripts might represent fusions of prokaryotic and eukaryotic genes.

In contrast to heterotrophic organisms, plants contain a large family of Trx and Trx-like proteins, although the reasons for their abundance remain unclear [106], [107]. We found that *Symbiodinium* species appear to possess an unexpectedly high number of Trx domain encoding genes comparable to plants, and in stark contrast to the substantially smaller number found in diatoms. The Trx superfamily proteins fulfill diverse cellular functions. These include the maintenance of cell homeostasis and the regulation of the redox state of the cell [108], [109]. They play key roles in the oxidative stress response [110], [111] and have been shown to be differentially expressed in response to high temperature, salinity, and ultraviolet radiation in corals [28], [112], [113]. Members of these groups are also involved in photosystem repair in response to ROS in plants and algae [114], [115]. Although the function of the large number of Trx containing genes can currently not be assessed, this high abundance might be indicative of a complex redox regulatory system in *Symbiodinium* as in plants [116]. The general similarity of the antioxidant gene repertoire of *Symbiodinium* and plants suggests that *Symbiodinium* might well be adapted to high oxidative stress. The comparably low amount of antioxidant genes found in both diatom species, which supposedly share more similarities with dinoflagellates than plants, raises the question whether *Symbiodinium* might have evolved a richer repertoire of antioxidant genes as an adaptation to its symbiotic lifestyle. More comprehensive transcriptome studies in other dinoflagellates are necessary to determine whether the features revealed in this study are specific to *Symbiodinium* or common among dinoflagellates.

This study greatly enhances the available sequence data for dinoflagellates in general, and *Symbiodinium* in particular. Our data highlight interesting aspects of the genetics of *Symbiodinium*, and provides the basis for deeper insights into dinoflagellate biology.

## Supporting Information

Figure S1 Relative fraction of all transcription factor domains in the *Symbiodinium* transcriptomes, all Dinoflagellate ESTs from the NCBI database, and other eukaryotes. Values shown were arcsine transformed. Searches were performed by using HMMER to query the Pfam models for DNA binding domains with an e-value cutoff of 1e−6.
(PDF)

Table S1 Cluster numbers and contigs and singlets contained in the clusters.
(XLS)

Table S2 Contigs and singlets of the actin gene family and their grouping into contigs (90% similarity).
(XLS)

Table S3 Histone genes and sequences.
(XLS)

Table S4 Distribution of contigs and singlets among KEGG categories and subcategories.
(XLS)

Table S5 Universal single-copy clusters of orthologous groups (COGs) that were used for transcriptome completeness analysis in this study. COGs not identified in *Symbiodinium* transcriptomes are boldfaced.
(XLS)

Table S6 Number of transcription factor domains found in *Symbiodinium* and other eukaryotes.
(XLS)

Table S7 Transcription factor domain counts in *Symbiodinium* CassKB8 and Mf1.05b, all dinoflagellate ESTs, *Plasmodium falciparum* and *P. vivax*, *Paramecium tetraurelia*, *Thalassiosira pseudonana*, *Arabidopsis thaliana*, *Drosophila melanogaster* and human.
(XLS)
