# Supplementary figures and images for: Symbiodinium Transcriptomes: Genome Insights into the Dinoflagellate Symbionts of Reef-Building Corals
Source: PLoS One. 2012 Apr 18;7(4):e35269. doi: 10.1371/journal.pone.0035269 (PMC3329448; doi:10.1371/journal.pone.0035269)

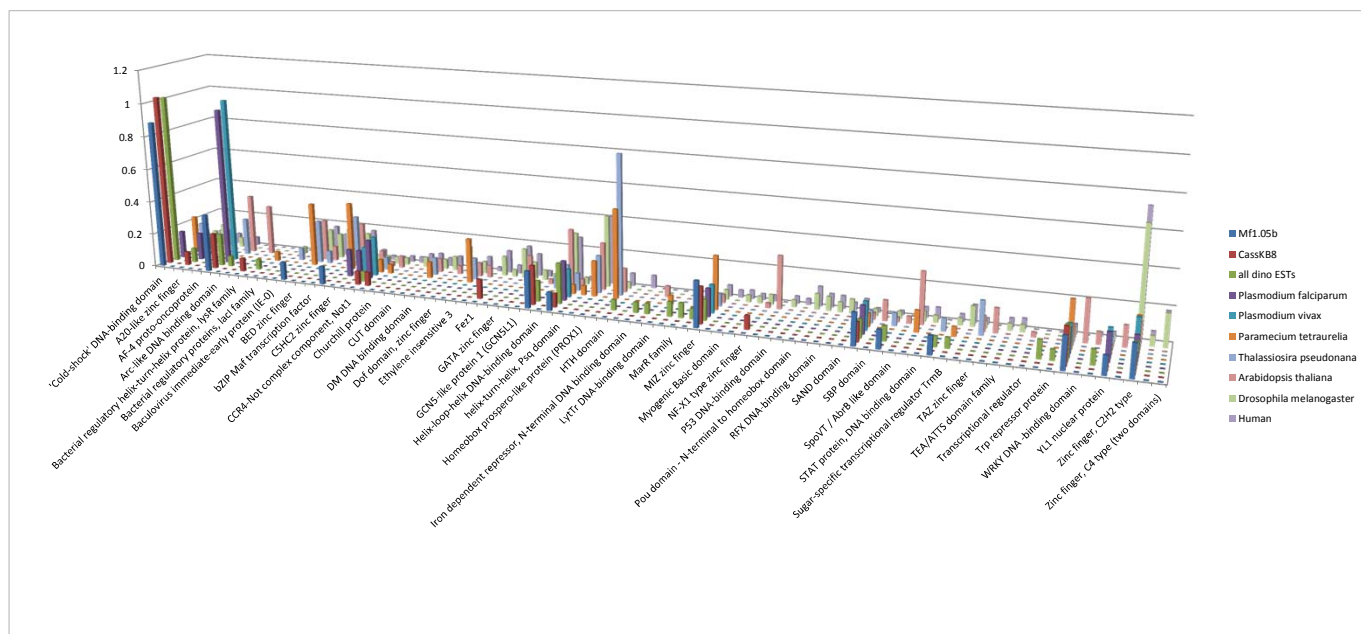

Supplement: Figure S1 — Relative fraction of all transcription factor domains in the Symbiodinium transcriptomes, all Dinoflagellate ESTs from the NCBI database, and other eukaryotes. Values shown were arcsine transformed. Searches were performed by using HMMER to query the Pfam models for DNA binding domains with an e-value cutoff of 1e−6. (PDF) [file pone.0035269.s001.pdf]
